# High* b*-Value Diffusion MRI to Differentiate Recurrent Tumors from Posttreatment Changes in Head and Neck Squamous Cell Carcinoma: A Single Center Prospective Study

**DOI:** 10.1155/2016/2865169

**Published:** 2016-06-07

**Authors:** Angela Acampora, Gaetana Manzo, Giacomo Fenza, Giuseppina Busto, Antonietta Serino, Andrea Manto

**Affiliations:** ^1^Department of Advanced Biomedical Sciences, Section of Radiology, University of Naples “Federico II”, Via Sergio Pansini 5, 80131 Naples, Italy; ^2^Department of Neuroradiology, Umberto I Hospital, Viale San Francesco 2, 84014 Nocera Inferiore, Italy; ^3^Department of Onco-Hematology, DEA Nocera-Pagani, Umberto I Hospital, Viale San Francesco 2, 84014 Nocera Inferiore, Italy

## Abstract

Recently DW-MR Imaging has shown promising results in distinguishing between recurrent tumors and posttreatment changes in Head and Neck Squamous Cell Carcinoma (HNSSC). Aim of this study was to evaluate the diagnostic performances of DWI at high* b*-value (*b* = 2000 s/mm^2^) compared to standard* b*-value (*b* = 1000 s/mm^2^) and ADC_ratio_ values (ADC_ratio_ = ADC_2000_/ADC_1000_ × 100) to differentiate recurrent tumors from posttreatment changes after treatment of HSNCC. 20 patients (16 M, 4 F) underwent MR Imaging between 2 and 16 months (mean 7) after treatment. Besides morphological sequences, we performed single-shot echo-planar DWI at* b* = 1000 s/mm^2^ and* b* = 2000 s/mm^2^, and corresponding ADC maps were generated (ADC_1000_ and ADC_2000_, resp.). By considering contrast-enhanced T1-weighted images as references, ROIs were drawn in order to evaluate mean ADC_1000_, ADC_2000_, and ADC_ratio_. The mean ADC_1000_ and ADC_2000_ in recurrent tumors were significantly lower than those in posttreatment changes (*P* = 0.001 and *P* = 0.016, resp.). Moreover, the mean ADC_ratio_ between the two groups showed a statistically significant difference (*P* = 0.002). Sensitivity, specificity, and accuracy of ADC_ratio_ were 82.0%, 100%, and 90%, respectively, by considering an optimal cutoff value of 65.5%. ADC_ratio_ is a promising value to differentiate between recurrent tumors and posttreatment changes in HNSCC and may be more useful than ADC_1000_ and ADC_2000_.

## 1. Introduction

Squamous cell carcinoma represents almost 90% of the head and neck tumors (HNSCC) and it shows different biological behaviors according to location [1].

Imaging techniques are commonly required in order to define tumor's locoregional extension [2]. Moreover MRI is increasingly becoming the preferred examination method as it provides additional information on tumor extension, muscles and lymph nodes involvement, and skull base and intracranial invasion. The role of MRI can therefore modify the clinical staging and consequently therapeutical approach, which is multidisciplinary with surgery, radiation therapy, and/or chemotherapy.

In these patients, however, even if treatment improves survival and quality of life, on the other hand, it can delay detection of residual or recurrent tumor in posttreatment follow-ups as surgery can modify anatomy and radiochemotherapy can result in edema, inflammation, fibrosis, and, sometimes, necrosis [3–5].

Conventional MR Imaging gives sometimes wrong information as postcontrast enhancement of a benign lesion may mimic a residual or a recurrent tumor, leading to an increased number of false positives and unnecessary surgical approaches. On the contrary, a recurrent tumor may not show enhancement after the administration of contrast medium.

Recently, DW (diffusion-weighted) MR Imaging has shown promising results. DWI is based on the Brownian motion of water protons, which is affected by the microstructure of tissue. Several promising studies have been reported on the usefulness of DWI in the discrimination between recurrent or residual tumor and posttreatment changes [6–8].

In recent years, high* b*-value (*b* > 1000 s/mm^2^) DWI was introduced along with improvements in MR Imaging gradient technology compared with standard* b*-value (*b* = 1000 s/mm^2^) DWI [9–11].

The aim of our present study was to evaluate the diagnostic performance of high* b*-value (*b* = 2000 s/mm^2^) DW sequences compared with standard* b*-value (*b* = 1000 s/mm^2^) DWI, and the ratio of ADC values from high and standard* b*-values for the differentiation between recurrent tumor and posttreatment changes after treatment (chemoradiotherapy and/or surgery) of HSNCC.

## 2. Materials and Methods

### 2.1. Population

A prospective study has been conducted between January 2013 and April 2014 by analysing 20 consecutive patients (16 M, 4 F, age range 41–81 years, mean age 60) with a previous (pathologically confirmed) diagnosis of Head and Neck Squamous-Cellular tumor (Table 1). All patients have been analysed in their follow-up after surgery and/or chemoradiotherapy after a strong suspicion of residual or recurrent tumor because of the recurrence of symptoms (Pt. numbers 5, 6, 7, 12, 13, 16, 19, and 20) or because of clinical suspicion at follow-up (e.g., palpable mass) (Pt. numbers 1, 2, 3, 4, 8, 9, 10, 11, 14, 15, 17, and 18). Primary tumor locations were nasopharynx (*n* = 10), tongue (*n* = 3), parotid gland (*n* = 3), oropharynx (*n* = 2), larynx (*n* = 1), and cheek (*n* = 1). All patients with nasopharynx tumors (Pt. numbers 1, 2, 3, 4, 5, 9, 11, 13, 17, and 18) were treated with 3 cycles of docetaxel, cisplatin, and 5-fluorouracil (TPF schedule) as induction chemotherapy followed by a subsequent concomitant chemoradiation.

As regarding patients with tongue cancer, one patient (Pt. number 20) was treated with surgery and then chemotherapy (cisplatin) and another one (Pt. number 6) with concomitant chemoradiotherapy while the third one underwent both a conservative surgical approach and right laterocervical lymph nodes dissection (II-III levels) followed by concomitant chemoradiation.

Patients affected by oropharynx cancer (Pt. numbers 12 and 15) were treated with chemotherapy and radiotherapy.

Those with ductal epidermoid cancer of the parotid gland (Pt. numbers 10, 14, and 19) were treated with parotidectomy followed by chemotherapy and radiotherapy.

The patient with a transglottic laryngeal cancer (Pt. number 7) was treated with chemotherapy and concomitant radiation therapy.

The one with a spinocellular cancer of the right cheek (Pt. number 8) had the resection of the lesion followed by locoregional radiotherapy.

Radiotherapy was performed with a linear multileaf accelerator and radiation field dimensions were decided by taking into account tumor extension, staging, and risk of lymph nodes involvement.

### 2.2. MR Imaging

All patients underwent head/neck area MR Imaging between 2 and 16 months (mean 7) after treatment by using a 1.5 T MR Imaging system (Philips Intera) with a surface head and neck synergy coil.

The study protocol included

- SE T1w axial (TR: 1300–2000 ms; TE: 15 ms; NSA: 2; section thickness: 4 mm; FOV: 220 × 220 mm; matrix: 224 × 157);
- TSE T2w axial (TR: 9900–14800 ms; TE: 120 ms; NSA: 2; section thickness: 4 mm; FOV: 220 × 220 mm; matrix: 224 × 157);
- STIR axial and coronal (TR: 1600 ms; TE: 23 ms; NSA: 2; section thickness: 4 mm; FOV: 220 × 220 mm; matrix: 336 × 235);
- SE T1w axial and coronal with fat suppression (TR: 800–3100 ms; TE: 15 ms; NSA: 2; section thickness: 4 mm; FOV: 220 × 220 mm; matrix: 368 × 256);
- SE T1w with fat suppression (TR: 800–3300 ms; TE: 15 ms; NSA: 2; section thickness: 4 mm; FOV: 220 × 220 mm; matrix: 368 × 258) after intravenous injection of 0.1 mmol/kg of gadoterate meglumine (Dotarem; Guerbet, Milan, Italy) in transverse, coronal, and sagittal planes.

EPI single-shot DWI sequences were obtained in the transverse plane before contrast agent injection both at standard* b*-values (*b* = 0 and* b* = 1000 s/mm^2^; TR: 8000 ms; TE: 89 ms; NSA: 1; section thickness: 4 mm; bandwidth: 1913 Hz/pixel; FOV: 240 × 240 mm; matrix: 112 × 89) and high* b*-values (*b* = 0 and* b* = 2000 s/mm^2^; TR: 9325 ms; TE: 107 ms; NSA: 4; section thickness: 4 mm; bandwidth: 1833 Hz/pixel; FOV: 240 × 240 mm; matrix: 112 × 89).

DWI data were acquired in 3 orthogonal directions (*X*,* Y,* and* Z*) and combined into a trace image. The average duration of DWI at standard* b*-value (*b* = 0 and* b* = 1000 s/mm^2^) was 58 sec while at high* b*-values (*b* = 0 and* b* = 2000 s/mm^2^) was 2 min and 20 sec.

The corresponding ADC maps were automatically derived from the following equation: ADC = ln⁡[*S*(*b*)/*S*(0)]/*b*, where* b* is the diffusion weighting factor (*b* = 1000 or 2000 s/mm^2^), and *S*(*b*) and *S*(0) are the signal intensities with and without diffusion-sensitizing gradients, respectively.

### 2.3. Image Analysis

MR images were reviewed on a PACS workstation monitor (Carestream Health).

ADC_ratio_ maps (ADC_ratio_ = ADC_2000_/ADC_1000_ × 100, where ADC_1000_ and ADC_2000_ are the ADC values of DWI obtained with* b* = 0 and 1000 s/mm^2^, and* b* = 0 and 2000 s/mm^2^, resp.) were generated by a specific software by use of pixel-by-pixel computation of ADC maps.

For each patient, two radiologists analysed both conventional MRI and diffusion-weighted (DW) sequences at the same time and recurrence versus posttreatment changes diagnosis was obtained by radiologists' concordance.

MRI examination was followed by biopsy in all patients in order to confirm the diagnosis.

Eight patients underwent surgical biopsy (Pt. numbers 5, 6, 7, 12, 13, 16, 19, and 20), 8 core biopsy (Pt. numbers 1, 2, 3, 4, 9, 11, 15, and 17), and 4 FNAB (Pt. numbers 8, 10, 14, and 19). Biopsy was performed 10–20 days after MRI examination (average time 15 days).

An expert pathologist confident with head and neck tumors analysed every sample.

#### 2.3.1. Qualitative Analysis: Conventional MRI and DW Sequences

The analysis included tumor site alterations such as high signal intensity on T2w and STIR sequences, tissue volume increase (focal or diffuse), mass effect, and enhancement pattern after contrast medium injection.

The increasing volume of focal areas of enhancement, at subsequent controls, was considered highly suspicious of tumor recurrence while volume reduction of the lesion or loss of growth at follow-ups was considered as posttreatment changes.

Regarding DW sequences, high signal intensity on high* b*-value images but low signal intensity on corresponding ADC maps were considered suggestive of recurrent tumor whereas high signal intensity on high-*b*-value images but generally high signal intensity on the corresponding ADC maps were considered as a result of T2 shine-through effect and therefore predictive of posttreatment changes.

#### 2.3.2. Quantitative Analysis: DW Sequences

In order to analyse ADC values in suspected areas, radiologists placed ROIs (regions of interest) on the axial ADC_1000_ maps with references of contrast-enhanced T1-weighted images obtained in 3 orthogonal planes. ROIs were drawn on the most representative section of the ADC map, in which the size of the tumor was the largest or the conspicuity of the lesion was the highest. ROI was drawn as large as the visible tumor was on that section of the ADC map corresponding to the contrast-enhanced T1-weighted images, trying to avoid necrotic portions and normal osseous structures.

Subsequently, ROIs were copied onto the corresponding ADC_2000_ and ADC_ratio_ maps, respectively.

The size of each ROI was also recorded, varying according to tumor extension (average size 189 ± 65 mm^2^).

### 2.4. Statistical Analysis

Statistical analysis was performed with SPSS Statistics 19.0 for Windows. For all statistical analyses, a 2-tailed *P* value of 0.05 was considered to indicate a statistically significant difference. The 2-tailed independent Student's* t*-test was used to compare mean ADC_1000_, ADC_2000_, and ADC_ratio_ values between the group with recurrent tumor and the group with post-treatment changes.

Student's* t*-test was also performed in order to compare mean ADC_2000_ values decreasing percentages versus ADC_1000_ ones between recurrent tumor group and posttreatment changes one.

A ROC (receiver operating characteristic) curve was drawn to investigate the optimal cutoff values for ADC_1000_, ADC_2000_, and ADC_ratio_ in order to obtain the best sensitivity, specificity, and accuracy in distinguishing between recurrent tumor and posttreatment changes.

## 3. Results

In our study, 11 patients were found to have a recurrent tumor and 9 posttreatment changes.

Conventional MRI sequences showed different degrees of anatomic changes after therapy, with a tissue asymmetric increased thickness as the most common. Suspected areas volume increase, together with a high signal intensity on T2w and STIR images and enhancement after contrast injection were found in 10 patients out of 11 with pathologically confirmed tumor and in 5 patients out of 9 with posttreatment changes (sensitivity 90%, specificity 44%, and accuracy 70%).

**Figure 1:**
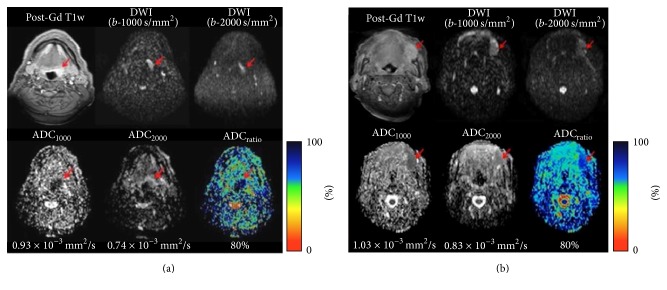
Representative MR images of recurrent tumors. (a) Patient number 12 (M, 68 yo) treated with chemoradiotherapy for a squamous cell carcinoma of the oropharynx. Postcontrast T1-weighted sequence shows an enhanced thickening of the left wall of oropharynx. (b) Patient number 20 (F, 74 yo) after surgery and radiotherapy for a squamous cell carcinoma of the tongue. Postcontrast T1-weighted sequence demonstrates an enhanced exophytic lesion of the left margin of the tongue. Recurrent tumors are characterized by high signal intensity on DWI at standard and high* b*-values and low signal intensity on the corresponding ADC maps. Mean ADC_1000_, ADC_2000_, and ADC_ratio_ were 0.93 × 10^−3^ mm^2^/s, 0.74 × 10^−3^ mm^2^/s, and 80% (a) and 1.03 × 10^−3^ mm^2^/s, 0.83 × 10^−3^ mm^2^/s, and 80% (b).

Recurrent tumors appeared as high signal intensity areas on DWI sequences and low signal ones in the corresponding ADC maps (Figure 1), except for one lesion that had a high signal intensity in the ADC maps; in fact this area showed a relatively high ADC_1000_ value (1.50 × 10^−3^ mm^2^/s) and intermediate ADC_2000_ and ADC_ratio_ values (0.85 × 10^−3^ mm^2^/s and 56%, resp.) and it was considered as a posttreatment change (Pt. number 19).

**Figure 2:**
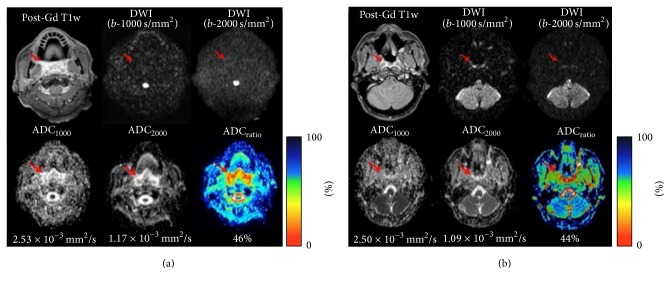
Representative MR images of posttreatment changes: (a) patient number 3 (M, 69 yo) after radiation therapy for an oropharynx cancer. Postcontrast T1-weighted sequence shows a diffuse and enhanced oropharyngeal thickening. Signal intensity is not increased on DW images at standard and high* b*-values. (b) Patient number 9 (M, 50 yo) after chemoradiotherapy for a nasopharyngeal cancer. Postcontrast T1-weighted sequence shows a right posterolateral thickening of nasopharynx. DW images at standard and high* b*-values depicted a mild high signal intensity (T2 shine-through effect). In both cases, posttreatment changes are characterized by high signal intensity on the corresponding ADC maps. Mean ADC_1000_, ADC_2000_, and ADC_ratio_ were 2.53 × 10^−3^ mm^2^/s, 1.17 × 10^−3^ mm^2^/s, and 46% (a) and 2.50 × 10^−3^ mm^2^/s, 1.09 × 10^−3^ mm^2^/s, and 44% (b).

Posttreatment changes showed high or low signal intensity on DWI sequences and high signal intensity on the corresponding ADC maps (Figure 2), except for two, which showed low/mixed signal intensity. Furthermore, their ADC_1000_ values were relatively low (1.40 × 10^−3^ mm^2^/s and 1.48 × 10^−3^ mm^2^/s resp.) while ADC_2000_ (0.86 × 10^−3^ mm^2^/s and 0.93 × 10^−3^ mm^2^/s, resp.) and ADC_ratio_ (61% and 63%, resp.) values were high and they were classified as recurrent tumors (Pt. numbers 1 and 14).

With information given by DWI sequences, MRI accuracy was 85%, with sensitivity and specificity of 90% and 78%, respectively.

In our study the smallest lesion found to be a recurrent tumor had a maximum diameter of 8 mm.

### 3.1. Comparison of ADC_1000_, ADC_2000_, and ADC_ratio_ between Patients with Recurrent Tumor and Patients with Posttreatment Changes

11 patients with residual/recurrent tumor had ADC_1000_ values between 0.93 × 10^−3^ mm^2^/s and 1.50 × 10^−3^ mm^2^/s with mean ADC_1000_ of 1.27 ± 0.22 × 10^−3^ mm^2^/s.

Nine patients with posttreatment changes had ADC_1000_ values between 1.40 × 10^−3^ mm^2^/s and 2.53 × 10^−3^ mm^2^/s with mean ADC_1000_ of 1.90 ± 0.44 × 10^−3^ mm^2^/s.

The mean ADC_1000_ between the two groups showed a statistically significant difference (*P* = 0.001).

ADC_2000_ values of recurrent tumor group ranged from 0.72 × 10^−3^ mm^2^/s to 1.05 × 10^−3^ mm^2^/s with a mean of 0.87 ± 0.13 × 10^−3 ^mm^2^/s while the ones in the group with posttreatment changes varied between 0.79 × 10^−3^ mm^2^/s and 1.44 × 10^−3^ mm^2^/s with a mean of 1.08 ± 0.21 × 10^−3^ mm^2^/s. The difference between the mean ADC_2000_ of the two groups was statistically significant (*P* = 0.016).

ADC_ratio_ values varied from 54% to 80% with a mean value of 69.5 ± 9.3% in the recurrent tumor group, while they varied from 46% to 65% with a mean value of 56.8 ± 6.3% in the posttreatment changes group.

The difference between the mean ADC_ratio_ of the recurrent tumor group and that of the posttreatment changes one was statistically significant (*P* = 0.002) (Tables 2 and 3).

ADC_2000_ values were significantly lower than ADC_1000_ ones (*P* < 0.001) with a decreasing percentage of 31 ± 9.7% in the recurrent tumor group and 42.6 ± 6.5% in the posttreatment changes group (Table 4).

A statistically significant difference was also found in the decreasing percentage of the mean ADC_2000_ versus ADC_1000_ between recurrent tumor group and posttreatment changes (31 ± 9.7% versus 42.6 ± 6.5%; *P* = 0.007).

### 3.2. Optimal Cutoff Values and Diagnostic Performances

By considering an optimal cutoff value of 1.53 × 10^−3^ mm^2^/s for the ADC_1000_ in order to distinguish between recurrent tumor and posttreatment changes, we obtained the higher accuracy of 90% (18/20), while sensitivity, specificity, PPV, and NPV were 100% (11/11), 78% (7/9), 85%, and 100%, respectively.

By considering an optimal cutoff value of 0.99 × 10^−3^ mm^2^/s for ADC_2000_ accuracy was 75% (15/20), while sensitivity, specificity, PPV, and NPV were 82% (9/11), 67% (6/9), 75%, and 75%, respectively.

For ADC_ratio_, the optimal cutoff value was 65.5%, and the accuracy was 90% (18/20), while sensitivity, specificity, PPV, and NPV were 82% (9/11), 100% (9/9), 100%, and 82%, respectively (Table 5).

## 4. Discussion

DW imaging is a functional MR Imaging technique that enables depiction and quantification of the Brownian motion of water molecules in vivo [12–14] therefore reflecting the variability of cellular tissue structure. The ability to investigate intracellular environment has led us to believe that DWI and the corresponding ADC values (apparent diffusion coefficient) can easily distinguish between neoplastic tissues and necrotic nonneoplastic ones. In 2001 Baur et al. analysed the role of diffusion-weighted imaging to differentiate tumor recurrences from posttherapeutical soft-tissue changes in musculoskeletal tumors, by qualitatively evaluating regions of interest's loss of signal on DW imaging; they found that the loss of signal was significantly higher for hygromas and muscles edematous changes compared to recurrent tumors [15]. Not many years later, Hein et al. demonstrated a statistically significant difference between ADC values of recurrent high-grade gliomas and posttreatment changes [16].

According to these encouraging results, several studies have been conducted to assess the role of DW sequences in distinguishing between recurrent tumor and posttreatment changes in head and neck squamous cell tumors. In 2010 King et al. showed how DW imaging enables detection of early pathologic changes after chemoradiotherapy in 50 patients with HNSCC and particularly found that ADC_1000_ value measured in a suspected area at follow-up is an important marker of recurrent tumor. Moreover, with a cutoff value of 1.40 × 10^−3^ mm^2^/s, the accuracy was 95% [6].

In a retrospective study on 33 patients (Hwang et al., 2013) with high suspicion of recurrent tumor after chemoradiotherapy and/or surgery, with DW imaging at standard* b*-values (0 and 1000 s/mm^2^) and high* b*-values (0 and 2000 s/mm^2^), the comparison of ADC_1000_, ADC_2000_, and ADC_ratio_ values between the two groups of patients (recurrent lesions versus posttreatment changes) depicted a statistically significant difference only for ADC_1000_ and ADC_ratio_ mean values in the two groups. By choosing a cutoff value of 1.46 × 10^−3^ mm^2^/s for ADC_1000_ and of 62.6% for ADC_ratio_ the diagnostic accuracy was 85% and 84.8%, respectively [11].

Our results demonstrate that ADC values derived from standard* b*-value DWI (1000 s/mm^2^) had a similar diagnostic performance compared with previous studies and that the mean ADC_1000_ in the posttreatment changes group was significantly higher than the one of the recurrent tumor group (1.90 ± 0.44 × 10^−3^ mm^2^/s versus 1.27 ± 0.22 × 10^−3^ mm^2^/s, resp.; *P* = 0.001).

The optimal cutoff value for the ADC_1000_ (1.53 × 10^−3^ mm^2^/s) to distinguish between recurrent tumor and posttreatment changes and the diagnostic accuracy (90%) is also related to previous studies (Table 6). These findings support the use of standard* b*-value DWI on posttreatment follow-up imaging of HNSCC as the mean values of the ADC_1000_ in the recurrent tumor group are significantly lower than those of the group with posttreatment changes.

According to Hwang et al.'s study, we observed a significant difference in the mean ADC_ratio_ between the recurrent tumor group and the posttreatment changes one (69.5 ± 9.3% versus 56.8 ± 6.3%, resp.; *P* = 0.002); with a cutoff value of 65.5% the diagnostic accuracy was 90% (Table 7).

Furthermore, contrary to Hwang et al.'s study, we also observed a statistically significant difference in the ADC_2000_ mean values between the two groups (0.87 ± 0.13 × 10^−3 ^mm^2^/s versus 1.08 ± 0.21 × 10^−3^ mm^2^/s, resp.; *P* = 0.016). With an ADC_2000_ cutoff value of 0.99 × 10^−3^ mm^2^/s, the accuracy was 75% (Table 8).

The two groups ADC values reflect the differences in histopathology and in water molecules distribution between tumors and posttreatment changes. Indeed, ADC is inversely related to tumor cellularity as the increase in cells number and in nucleus dimensions in malignant lesions leads to limitation of diffusion of water molecules and, consequently, to lower ADC values.

On the other hand, decreased cellularity and the presence of edema and inflammatory changes in posttreatment modification are related to higher ADC values.

According to our results, ADC values have also a substantial decrease by increasing the* b*-value over 1000 s/mm^2^.

DeLano et al. subjected 50 patients to brain diffusion-weighted imaging with* b*-values of 0, 1000, 2000, 2500, 3000, and 3500 s/mm^2^. For 6 patients, apparent diffusion coefficient maps were generated at* b*-values of 0 and at* b*-values of 1000, 2000, and 3000 s/mm^2^. Quantitative assessments were made in multiple regions of interest in gray and white matter and, for some ROI, the ADC decreased approximately of 30% to 35% for* b*-values between 1000 and 3000 s/mm^2^. These data indicate that if the relationship between MR signal and* b*-values would have been monoexponential, the ADC value should remain constant as the* b*-value increases. A likely explanation is that apparent diffusion is more accurately represented by a biexponential relation [17].

Both in animal and human models, rapidly decaying and slowly decaying diffusion components have been described. At low* b*-values, signal intensity is dominated by a fast diffusion component, *D*_fast_, whereas, at higher* b*-values, signal intensity is contributed largely by the slower component of diffusion, *D*_slow_. Intracellular and extracellular water are considered to correspond to slow and fast diffusion components, respectively, even if not exactly equal to them.

The increasing of intracellular water due to hypercellularity of a recurrent tumor and the reduction of the extracellular space can thus explain the more significant influence of the slow diffusion component on DWI sequences; on the contrary, posttreatment changes edema is related to the predominance of the extracellular water and, consequently, of the fast diffusion component [18].

In our study ADC_1000_ and ADC_2000_ maps were obtained from DWI sequences at two* b*-values (0 and 1000 s/mm^2^; 0 and 2000 s/mm^2^, resp.) with a total scan time of 3 min and 20 sec.

We hypothesized that, by increasing the* b*-value from 1000 s/mm^2^ to 2000 s/mm^2^, the decrease in ADC value would reflect the proportion between slow and fast diffusion components in suspected areas.

The analysis of the two groups (recurrent tumor and posttreatment changes) showed a lower decreasing percentage of the mean ADC_2000_ compared to the mean ADC_1000_ in recurrent tumor group (31 ± 9.7% versus 42.6 ± 6.5%; *P* = 0.007) and a higher ADC_ratio_ (69.5 ± 9.3% versus 56.8 ± 6.3%; *P* = 0.002). We suggest that the lower decrease of ADC values of recurrent lesions by increasing the* b*-value from 1000 to 2000 s/mm^2^ can be easily explained by the higher cellularity thus reflecting the greater influence of the slow diffusion component. Furthermore, in tumors, the greater the proportion of slow diffusion component becomes, the higher the ADC_ratio_ increases, and, for this reason, the latter might become an alternative biomarker for higher cellularity and it might also simplify the detection of recurrent tumor by visual inspection in the ADC_ratio_ maps.

Our results are in agreement with those of earlier studies.

In the present study, qualitative and quantitative analysis of DWI sequences and the corresponding ADC maps revealed one false negative and two false positives.

Signal intensity on diffusion-weighted images is influenced by two factors: water diffusibility and the intrinsic T2 properties of the tissue being examined. In tissues with very long T2 relaxation times, the strong T2 signal may be mistaken for restricted diffusion, a phenomenon known as “T2 shine-through effect.” The easiest way to distinguish between restricted diffusion and T2 shine-through is to generate an ADC map, on which the former appears as an area of low signal intensity (low ADC values) and the latter as a high-signal-intensity area [19].

Therefore, high signal intensity on high-*b*-value images and low signal intensity on corresponding ADC maps were considered suggestive of recurrent tumor whereas high signal intensity on high-*b*-value images and generally high signal intensity on the corresponding ADC maps were considered as a result of T2 shine-through effect and therefore predictive of posttreatment changes.

The relatively high ADC values of the recurrent tumor wrongly classified as posttreatment changes may be correlated with the presence of edema and small areas of postradiation colliquative necrosis as well reported by Matzek et al. in a previous study on oropharynx squamous cell carcinoma [20]. As concerns the two false positives, they underwent radiotherapy in the previous year. Nomayr et al. reported that the first visible effect in the first 6 months after irradiation is edema together with fibroinflammatory reaction and increasing permeability due to damage of vessels endothelium. Later on (6–24 months after irradiation) there is the regression of inflammatory edema and the increasing in fibrous reaction, which shows a restricted diffusion [4].

The choice of regions of interest (ROIs) places an important role in order to reduce pitfalls in the evaluation of mean ADC values. Firstly, ROIs should be drawn manually and no necrosis areas should be included; secondly, the more heterogeneous the lesion, the more the ROIs that should be considered from which a mean value should be extrapolated.

However, partial volume artifacts and areas of micronecrosis cannot be avoided during measurement of ADC values therefore still representing a sampling source of errors [21]. The overlapping of ADC values could be associated with partial volume artifacts due to structures sizes, wrongly low ADC values can be obtained with fibrosis, while wrongly high ADC values can be depicted in necrotic areas of recurrent tumors.

DWI single-shot echoplanar sequence (EPI) was performed for qualitative and quantitative analysis. It has a time resolution of less than 200 ms, thus guaranteeing data acquisition at different* b*-values in a relatively short total scan time. However its weakness is its extreme sensitivity to susceptibility artifacts consequently producing more geometrical distortion artifacts with the increase of* b*-values.

The head and neck region is particularly difficult for performing DW imaging acquisitions because it is very heterogeneous, containing a variety of tissues that include fat, muscle, air, glandular tissue, and bone. It also has very low acquirable signal (from muscle and suppressed fat) and a strongly changing geometric shape. This can yield images with a very low signal-to-noise ratio and strong susceptibility artifacts from many air tissue boundaries, as well as from metallic surgical implants and dental fillings. Moreover, head and neck area is subject to a number of movement-related problems [22–24].

One limitation of our study concerns patients' enrollment as the HNSCC population was quite heterogeneous (various locations and pathologic condition, different treatment modalities, and timing of imaging) and this heterogeneity has made the homogenization of our results difficult.

In our opinion, however, lesion location should not have a significant impact on ADC values whereas the standardization of imaging timing, which could theoretically have an influence on results, is difficult to apply.

Future studies on a less heterogeneous population are required to better analyze the role of these variables on ADC values.

## 5. Conclusions

The present prospective study on HNSCC patients treated with surgery and/or chemoradiotherapy has shown high accuracy of DW-MR Imaging in distinguishing between recurrent tumor and posttreatment changes. We also suggest that ADC measurement both at standard and high* b*-values is required to assess slow diffusion component, which is directly proportional to intracellular water. We suggest that the ADC_ratio_ calculated from ADC_1000_ and ADC_2000_ is a promising value to differentiate between recurrent tumor and posttreatment changes in HNSCC and may be more useful than ADC_1000_ and ADC_2000_ alone.

High* b*-value DWI of head and neck region is technically feasible and requires a relatively short additional scan time but should always be analysed by taking into account morphological sequences in order to correctly localize lesions. However, larger studies are required for the standardization of this imaging to improve single patient application.

## Figures and Tables

**Table 1:** Diagnosis and staging, therapy, and interval between treatment and imaging of 20 patients affected by Head and Neck Squamous Cell Carcinoma (HNSCC).

Patient	Age/sex	Diagnosis	Therapy	After treatment imaging (months)
1	64/M	Ca nasopharynx T_4a_N_1_M_0_	CTRT	13
2	54/F	Ca nasopharynx T_4a_N_2b_M_0_	CTRT	6
3	69/M	Ca nasopharynx T_3_N_1_M_0_	CTRT	8
4	50/M	Ca nasopharynx T_3_N_1_M_0_	CTRT	7
5	51/M	Ca nasopharynx T_4a_N_1_M_0_	CTRT	10
6	68/M	Ca tongue T_4a_N_2b_M_0_	CTRT	5
7	66/M	Ca larynx T_4a_N_2a_M_0_	CTRT	9
8	79/M	Ca cheek T_3_N_0_M_0_	Surgery + RT	10
9	50/M	Ca nasopharynx T_3_N_1_M_0_	CTRT	3
10	50/M	Ca parotid gland T_2_N_2a_M_0_	Surgery + CTRT	10
11	46/M	Ca nasopharynx T_4a_N_2b_M_0_	CTRT	8
12	68/M	Ca oropharynx T_2_N_1_M_0_	CTRT	5
13	55/M	Ca nasopharynx T_4b_N_2c_M_0_	CTRT	4
14	65/F	Ca parotid gland T_2_N_2a_M_0_	Surgery + CTRT	16
15	48/M	Ca oropharynx T_2_N_1_M_0_	CTRT	2
16	81/M	Ca tongue T_4a_N_2b_M_0_	Surgery + CTRT	4
17	77/F	Ca nasopharynx T_3_N_1_M_0_	CTRT	6
18	41/M	Ca nasopharynx T_4b_N_2c_M_0_	CTRT	8
19	53/M	Ca parotid gland T_2_N_2b_M_0_	Surgery + CTRT	5
20	74/F	Ca tongue T_1_N_0_M_0_	Surgery + CT	9

**Table 2:** ADC_1000_, ADC_2000_, and ADC_ratio_ range of values.

	Range ADC_1000_	Range ADC_2000_	Range ADC_ratio_
Tumor recurrence	(0.93–1.50) × 10^−3^ mm^2^/s	(0.72–1.05) × 10^−3^ mm^2^/s	54–80%
Posttreatment changes	(1.40–2.53) × 10^−3^ mm^2^/s	(0.79–1.44) × 10^−3^ mm^2^/s	46–65%

**Table 3:** ADC mean values of suspected areas.

	Value (mean ± SD)	Value (mean ± SD)	*P* value
	Recurrent tumor	Posttreatment changes	
ADC_1000_ (×10^−3^ mm^2^/s)	1.27 ± 0.22	1.90 ± 0.44	0.001
ADC_2000_ (×10^−3^ mm^2^/s)	0.87 ± 0.13	1.08 ± 0.21	0.016
ADC_ratio_ (%)	69.5 ± 9.3	56.8 ± 6.3	0.002

**Table 4:** ADC_1000_ and ADC_2000_ mean values and decreasing percentage.

	ADC_1000_	ADC_2000_	*P* value	Decreasing percentage
Recurrent tumor	1.27 ± 0.22	0.87 ± 0.13	<0.001	31 ± 9.7%
Posttreatment changes	1.90 ± 0.44	1.08 ± 0.21	<0.001	42.6 ± 6.5%

**Table 5:** Diagnostic performances and cutoff values of ADC_1000_, ADC_2000_, and ADC_ratio_.

	Cutoff value	Sensitivity	Specificity	PPV	NPV	Accuracy
ADC_1000_	1.53 × 10^−3^ mm^2^/s	100%	78%	85%	100%	90%
ADC_2000_	0.99 × 10^−3^ mm^2^/s	82%	67%	75%	75%	75%
ADC_ratio_	65.5%	82%	100%	100%	82%	90%

**Table 6:** ADC_1000_ cutoff value and diagnostic performances compared to previous studies.

Abdel Razek et al. [7]—32 Pt.	1.30 × 10^−3^ mm^2^/s	85%	90%	87%
Vandecaveye et al. [8]—26 Pt.	1.30 × 10^−3^ mm^2^/s	95%	95%	95%
King et al. [6]—20 Pt.	1.40 × 10^−3^ mm^2^/s	80%	100%	90%
Hwang et al. [11]—33 Pt.	1.46 × 10^−3^ mm^2^/s	85%	85%	85%
*Present study—20 Pt.*	1.53 × 10^−3^ mm^2^/s	100%	78%	90%

**Table 7:** Comparison of ADC_ratio_ cutoff value and diagnostic performances between the present study and Hwang et al.'s study [11].

	ADC_ratio_ cutoff value	Sensitivity	Specificity	Accuracy
Hwang et al. [11]—33 Pt.	62.6%	95%	69.2%	84.8%
*Present study*—*20 Pt*.	65.5%	82%	100%	90%

**Table 8:** Comparison of ADC_2000_ cutoff value and diagnostic performances between the present study and Hwang et al.'s study [11].

	ADC_2000_ cutoff value	Sensitivity	Specificity	Accuracy
Hwang et al. [11]—33 Pt.	—	—	—	—
*Present study*—*20 Pt.*	0.99 × 10^−3^ mm^2^/s	82%	67%	75%
